# An efficient microinjection method to generate human anaplasmosis agent *Anaplasma phagocytophilum*-infected ticks

**DOI:** 10.1038/s41598-020-73061-9

**Published:** 2020-09-29

**Authors:** Vikas Taank, Ellango Ramasamy, Hameeda Sultana, Girish Neelakanta

**Affiliations:** 1 Department of Biological Sciences, Old Dominion University, Norfolk, VA USA; 2 Center for Molecular Medicine, Old Dominion University, Norfolk, VA USA; 3 Department of Biological Sciences, Center for Molecular Medicine, College of Sciences, Old Dominion University, Norfolk, VA 23529 USA

**Keywords:** Microbiology, Bacteriology

## Abstract

Ticks are important vectors that transmit several pathogens including human anaplasmosis agent, *Anaplasma phagocytophilum*. This bacterium is an obligate intracellular rickettsial pathogen. An infected reservoir animal host is often required for maintenance of this bacterial colony and as a source for blood to perform needle inoculations in naïve animals for tick feeding studies. In this study, we report an efficient microinjection method to generate *A. phagocytophilum*-infected ticks in laboratory conditions. The dense-core (DC) form of *A. phagocytophilum* was isolated from in vitro cultures and injected into the anal pore of unfed uninfected *Ixodes scapularis* nymphal ticks. These ticks successfully transmitted *A. phagocytophilum* to the murine host. The bacterial loads were detected in murine blood, spleen, and liver tissues. In addition, larval ticks successfully acquired *A. phagocytophilum* from mice that were previously infected by feeding with DC-microinjected nymphal ticks. Transstadial transmission of *A. phagocytophilum* from larvae to nymphal stage was also evident in these ticks. Taken together, our study provides a timely, rapid, and an efficient method not only to generate *A. phagocytophilum*-infected ticks but also provides a tool to understand acquisition and transmission dynamics of this bacterium and perhaps other rickettsial pathogens from medically important vectors.

## Introduction

In the Northeastern part of the United States, blacklegged *Ixodes scapularis* ticks are the important vectors for human anaplasmosis agent *Anaplasma phagocytophilum*^1,2^. Several other *Ixodes* species in North America, Europe and East Asia also serves as main vectors for this bacterium^1–4^*.* In addition, *A. phagocytophilum* DNA was detected in *I. ovatus,* suggesting that these ticks could also act as primary vectors for this bacterium^5^. In the United States, ticks acquire *A. phagocytophilum* through blood meal during feeding on an infected reservoir hosts such as *Peromyscus leucopus* (white-footed mice), *Procyon lotor* (raccoons), *Sciurus carolinensis* (gray squirrels), *Urocyon cinereoargenteus (*gray foxes), and *Neotamias ochrogenys* (redwood chipmunks)^2,6^. Ticks have four stages in their life cycle, starting from the order eggs-larvae-nymphs-adults^7^. Except eggs, larvae and nymphal ticks require a blood meal to develop to the next stage, and adult female ticks require a blood meal to mate with an adult male ticks and lay eggs^7^. Upon entry into ticks, *A. phagocytophilum* is transstadially maintained in different molting stages from larvae to adult stage^1,2,6–8^. Various strains of *A. phagocytophilum* were detected in different regions of the world in ticks and diverse animals^9–11^. With the exception in *Dermacentor albipictus,* transovarial transmission of *A. phagocytophilum* variants has not been observed in other group of ticks^12^.

Humans get infected with *A. phagocytophilum* upon a bite with an infected tick^13^. The general clinical manifestations of *A. phagocytophilum* infection in humans include fever, thrombocytopenia, malaise, myalgia, leukopenia, headache, and increased serum aminotransferase liver enzyme activity that suggest mild injury to the liver^14^. The murine models with *A. phagocytophilum* infection have been noted to partially mimic the disease in humans and animals, including kinetics of infection, cytokine responses, histopathological observations similar to those observed in infected humans and other animals^15^. Different murine models such as BALB/c, C57BL/6J, C3H/HeJ, and C3H/HeN mice have been routinely used to study infection kinetics of human anaplasmosis and/or dynamics of *A. phagocytophilum* acquisition and transmission from mice-tick or tick-mice passages^15–26^. The *A. phagocytophilum* loads declines in these animals as the infection progress^27^. However, immunodeficient animals such as SCID mice can retain *A. phagocytophilum* infection up to 90 days^27,28^. Needle inoculation of naïve mice with *A. phagocytophilum* often requires use of infectious blood (from immunodeficient mice) as an inoculum^20,21,27^. Therefore, continuous maintenance of *A. phagocytophilum* in immunodeficient animals is expensive but required for the source of infectious inoculum to infect naïve mice.

In mammalian hosts, *A. phagocytophilum* primarily infects neutrophils where it resides inside an inclusion^29^. In both mammalian and tick cells, the developmental stages of *A. phagocytophilum* involve the formation of two morphotypes, a larger reticulate form (RC), and a smaller dense-core (DC) form^30,31^. It has been noted that only DC forms binds and enters the mammalian cells but both DC and RC forms can bind and enter tick cells^30,31^. Upon entry into mammalian cells, such as human leukemia cell line, HL-60, the DC forms transit to RC form within 12 h post infection and these forms initiate replication^31^. Inclusions with RC form can be observed by 24 h post infection of HL-60 cells^31^. By 36 h, inclusion with both DC and RC forms were evident^31^. The DC forms were ready by this time to re-infect the same host cell or infect naïve cells. *Anaplasma phagocytophilum* colonizes tick salivary glands and establishes persistent infection^32^. However, the presence of *Anaplasma* morphotypes in the tick tissues remains to be studied.

To generate *A. phagocytophilum* infected ticks, naïve mice such as C3H/HeN have to be first infected with infectious blood obtained from SCID and RAG^−/−^ mice or HL-60 cultures and naïve ticks (larvae or nymphs) are then allowed to feed on these infected-mice. Repleted larval or nymphal ticks can be molted to next stage to generate unfed *A. phagocytophilum*-infected nymphal or adult ticks, respectively. Even though this method is feasible, it requires enormous budget and more time for maintenance of these mice for longer duration. In this study, we report a timely, rapid, and an efficient method of microinjection of *A. phagocytophilum* into the anal pore of unfed nymphal ticks. This study not only provides a new method to generate *A. phagocytophilum*-infected ticks but also provides an efficient tool to study acquisition and transmission dynamics of this and perhaps other rickettsial pathogens of medical importance.

## Methods

### Bacterial isolate and preparation of host cell-free *A. phagocytophilum* dense core (DC) form

*Anaplasma phagocytophilum* isolate NCH-1, obtained from BEI resources, Centers for Disease Control and Prevention (CDC), was used in all studies involving in vivo work with ticks and mice; wherever necessary, NCH-1 isolate is referred as *A. phagocytophilum*. Maintenance of *A. phagocytophilum* was carried out in HL-60 (ATCC, CCL-240) cells as described^33^. Host cell-free *A. phagocytophilum-*DC was isolated from confluent > 70% HL-60 infected cells by sonication followed by differential centrifugation as described^33^. Briefly, infected HL-60 cells were centrifuged at 2300 g for 10 min at 4 °C and the pellet was re-suspended in 1× phosphate-buffered saline (PBS), followed by sonication (8 s bursts with 8 s resting periods) on ice and then passaged 6–8 times through 27 ½ gauge syringe needle. This sonicated lysate was processed further for differential centrifugation to separate the bacterial DC form from the host cells that included 750 g spin for 5 min to pellet host cell debris and centrifuging the supernatant again at 1000 g for 5 min. The supernatant was further centrifuged at 2300 g for 10 min. The pellet obtained is composed of host cell-free *A. phagocytophilum*-DC^33^. 1× PBS was added to resuspend this pellet and used for further analysis. Wherever necessary *A. phagocytophilum*-DC is referred as *Ap*-DC.

### Measurement of *A. phagocytophilum* DC particle size and number using microfluidic resistive pulse sensing (MRPS)

MRPS is based on coulter principle using a microfluidic cartridge and can be utilized to analyze particle sizes from 50 to 2000 nm in a solution^34,35^. We performed MRPS (nCS1, Spectradyne LLC, USA) on host cell-free *A. phagocytophilum*-DC to determine the size and concentration of DC particles. Infection in HL-60 cells was maintained as described^33^. Briefly, two cell culture flasks with 10 ml each containing approximately 1 × 10^6^/ml infected HL-60 cells were centrifuged and processed for DC form isolation as described in the previous section. Equal number of uninfected HL-60 cells was processed as control group. Final DC pellet was re-suspended in 500 μl of 1 × PBS. Control tube used for processing uninfected HL-60 cells was also treated with the same volume of 1 × PBS. Three microliters from both suspensions were processed for MRPS Spectradyne measurements. All samples were measured at − V = − 5.0 V and + V = + 6.0 V using factory calibrated TS-2000 cartridge: ~ 250–2000 nm particle size range. MRPS Spectradyne measurements are plotted to show particle size (concentration/ml) on Y-axis and particle diameter (nm) on the X-axis (Fig. 2, Supplementary Fig. S1). The ± numbers actually mean counting error. There are 2 error numbers, the first is negative error and the second is positive error. The values are calculated based on Poisson-distributed statistics, and assumption that particles arrive at the nano-constriction in a purely random fashion (i.e. no particle event is related to another, they all occur independently). The statistical counting error was calculated based upon 1/√(N) (1 divided by square root of N), where N is the number of particles counted. When using Resistive Pulse Sensing to make particle measurements there is always electrical noise in the system. All the blue dots (Supplementary Fig. S1) that go across bottom of the transit time scatterplot are purely noise and they were excluded from the analysis.

### Mice and ticks

**Figure 1:**
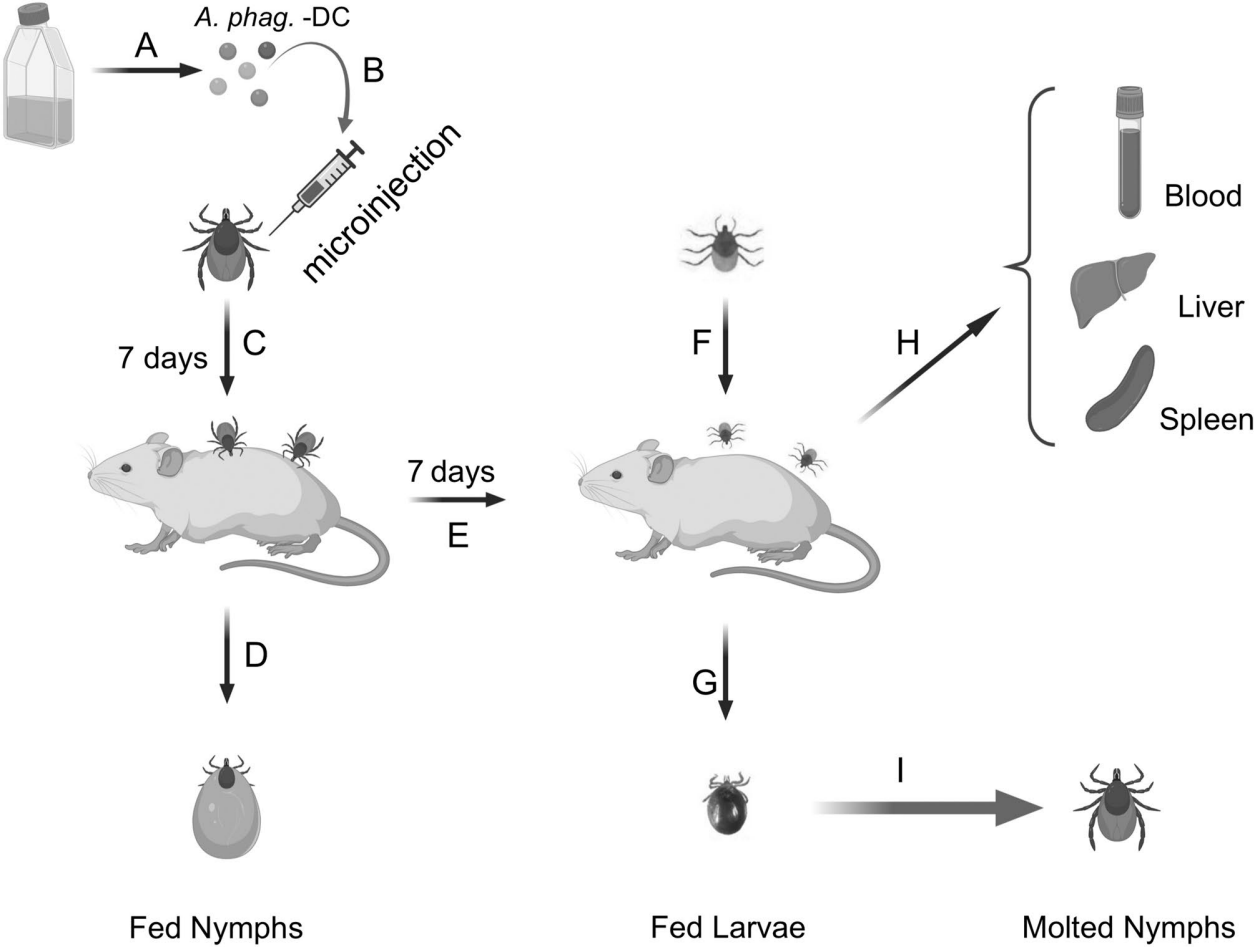
Schematic representation of microinjection of *A. phagocytophilum*-DC forms in *Ixodes scapularis* ticks. (**A**) Cell-free *A. phagocytophilum*-DC was isolated after sonication followed by differential centrifugations from infected HL-60 cells. (**B**) 1/100-fold dilutions of *Ap*-DC was prepared and microinjected into unfed uninfected nymphal ticks and ticks were incubated for 7 days. (**C**) Recovered ticks were infested on naïve mice. (**D**) Fully engorged/repleted ticks were collected. (**E**) The mice were housed for seven days. (**F**) Unfed larvae were allowed to feed on mice previously infected with *Ap*-DC injected nymphs. (**G**) Fully engorged/repleted larval ticks were collected. (**H**) After larval repletion, mice tissues (blood, spleen and liver) were collected. (**I**) Fully engorged larvae were allowed to molt into nymphs. This image was Created with BioRender.com.

Laboratory reared *I. scapularis* (larvae and nymphs) ticks obtained from a continuously maintained tick colony from BEI resources/Center for Disease Control and Prevention (CDC) or National Tick Research and Education Resource, Oklahoma State University were used in this entire study. C3H/HeN mice (female mice, 4–6 weeks old, CharlesRiver Laboratories, USA) were used in all animal experiments in this study. Uninfected nymphs were generated by feeding larvae on naïve mice and were allowed to molt into nymphs. Tick rearing was conducted in an incubator at 23 ± 2 °C with 90–95% relative humidity and a 14/10 h light/dark photoperiod regiment, as described in our previous studies^22,25,26^. DC-injected (via anal pore microinjection) unfed nymphal ticks (freshly molted) were fed on naïve C3H/HeN mice to transmit *A. phagocytophilum* from ticks to mice (Fig. 1). Upon repletion, these fed nymphal ticks were processed for DNA extractions followed by PCR to detect *A. phagocytophilum* loads. After day seven-post infection in C3H/HeN mice, larvae were allowed to feed on the same mice (generated by feeding with DC-injected ticks) for pathogen acquisition into ticks (Fig. 1). Upon repletion, these fed larvae were processed for DNA extraction, followed by PCR to detect *A. phagocytophilum* loads. After larval repletion, mice were euthanized and blood, liver and spleen were collected and processed for DNA extractions, followed by PCR to detect *A. phagocytophilum* (Fig. 1). In addition, a group of repleted larval ticks that were fed on DC-injected-tick-mediated infected mice were allowed to molt to nymphs (Fig. 1). DNA was extracted from molted nymphs and processed for PCR to detect bacterial loads.

### Ethics statement

All animal work in this study was carried out in strict accordance with the recommendations in the Guide for the Care and Use of Laboratory Animals of the National Institute of Health. The Old Dominion University Institutional Animal Care and Use Committee (IACUC, Animal Welfare Assurance Number: A3172-01) approved protocols 16-017 and 19-009 were used in mice experiments. Acepromazine Maleate injection, USP (2 μl from 10 mg/ml stock/30 g mouse) tranquilizer was administered to the animals prior to handling to minimize anxiety and/or discomfort, and all efforts were made to minimize suffering.

### Tick microinjections

The Eppendorf FemtoJet microinjection system was used in this study. Microinjections into ticks were performed as previously described^36^. The DC preparation (as described in an earlier methods section) from *A. phagocytophilum*-infected HL-60 was tenfold serially diluted twice and the diluted solution was used for microinjections into ticks. Micoinjections were performed into the anal pore of uninfected unfed nymphs (~ 4.2 nl/tick). During microinjection ticks were temporarily immobilized on a double-sided tape. Microinjected ticks were incubated at room temperature for seven days for acclimatization in an incubator at 23 ± 2 °C with 90–95% relative humidity and a 14/10 h light/dark photoperiod. After 7 days of incubation, DC-microinjected ticks were fed on naïve C3H/HeN mice to transmit the pathogen. Approximately, out of 20 ticks that were placed on mice, 8–10 DC-microinjected ticks successfully fed and repleted from each mouse. Repleted ticks were processed for DNA extraction followed by quantification of *A. phagocytophilum p44* loads by QRT-PCR/PCR using oligonucleotides as mentioned in Table S1. The PCR conditions for QRT-PCR/PCR are mentioned in the QRT-PCR and PCR analysis section.

### Isolation of DNA, total RNA and cDNA synthesis

In non-quantitative polymerase chain reaction (PCR) and Quantitative Real-Time-PCR (QRT-PCR) experiments, DNA was used to detect presence or absence of bacteria. For reverse-transcription polymerase chain reaction (RT-PCR), RNA was used to detect presence or absence of bacterial transcripts. To detect bacteria loads in ticks, genomic DNA from *A. phagocytophilum*-infected (DC-injected) unfed or fed nymphs or fed larvae or molted unfed nymphs and mice samples were extracted using DNeasy Blood and tissue kit (QIAGEN) and processed for PCR with primers specific for *A. phagocytophilum p44* gene as mentioned in Table S1. As an internal control*, I. scapularis* ribosomal 5.8s rRNA was analyzed in the same DNA samples using oligonucleotides as mentioned in Supplementary Table S1. Total RNA from *A. phagocytophilum* DC was generated using the Aurum Total RNA mini kit (Bio-Rad, USA) and following the manufacturer instructions. During RNA extraction, on-column DNase I digestion was performed as per manufacturer’s recommendation. The eluted RNA was then converted to cDNA using BioRAD cDNA synthesis kit (BioRAD, USA). The generated cDNA was used as a template for the detection of *A. phagocytophilum* transcripts (*dnaA* and *dnaK*) using the oligonucleotides shown in Supplementary Table S1. In DNA samples extracted from murine blood and tissue samples, bacterial burden was quantified by normalizing the level of *A. phagocytophilum p44* to mice beta-actin levels using oligonucleotides mentioned in Table S1.

### QRT-PCR and PCR analysis

QRT-PCR was performed as described^37^ using CFX96 QRTPCR machine (BioRad, USA), iQ-SYBR Green Supermix (BioRad, USA) and with DNA samples isolated from murine tissues. In QRT-PCR reactions, the standard curve for each gene fragment was generated using tenfold serial dilutions starting from 1 ng to 0.00001 ng of known quantities of respective fragments. For standard preparation, initial DNA concentration was measured by taking optical density reading in TECAN plate reader (TECAN, USA). After measurements of concentrations, tenfold serial dilutions were made to prepare various standards. PCR reactions were performed with Taq DNA polymerase (New England Biolabs Inc., USA). The PCR conditions were modified from^38^ and were used for both QRT-PCR and PCR. The conditions are as follows: 95 °C for 5 min initial denaturation followed by 95 °C for 1 min, 55 °C for 1 min, and 72 °C for 2 min for 40 cycles with final extension of 72 °C for 7 min. For RT-PCR, following conditions were used: 94 °C for 2 min 30 s initial denaturation followed by 94 °C for 30 s, 56 °C for 30 s, and 72 °C for 30 s for 34 cycles with final extension of 72 °C for 10 min. PCR or RT-PCR products were analyzed on 1.5% agarose gels stained with ethidium bromide. Internal quality control included parallel PCR amplification of no template control (ntc) and positive control (+, previously sequenced fragment) along with the samples. PCR products obtained from *A. phagocytophilum* specific oligonucleotides from every experimental group including murine blood, DC-injected fed nymphs (from transmission experiment), fed larvae (from acquisition experiment) and molted nymphs (from transstadial transmission experiment), were gel purified and sequenced at Eurofins (USA).

### Statistics

Statistical significance in the data sets was analyzed using GraphPad Prism6 software (https://www.graphpad.com/) and Microsoft Excel 2010 (https://www.microsoft.com). For data to compare two means, the non-paired Student’s *t*-test was performed. P values of < 0.05 were considered significant in all analyses. Wherever necessary, statistical test and P values used are reported.

## Results

### Analysis of particle size and concentration of *A. phagocytophilum* DC form

**Figure 2:**
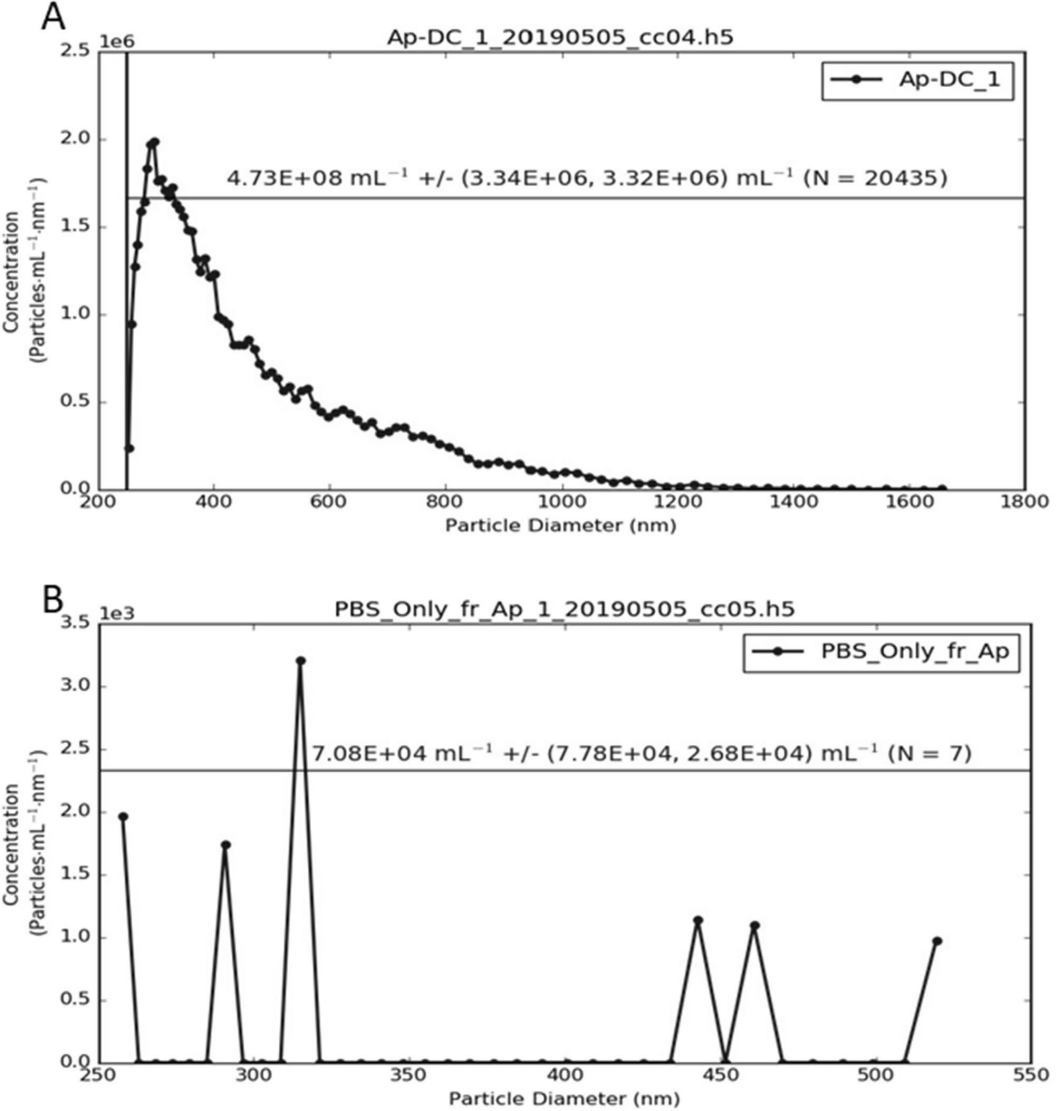
Concentration and particle size measurement of *Ap*-DC using Spectradyne device. (**A**) Microfluidics resistive pulse sensing (MRPS) using Spectradyne instrument (nCS1, Spectradyne LLC, USA) was used to determine the size distribution and concentration of isolated *Ap*-DC (**A**) and PBS control (**B**). TS-2000 cartridge was used to perform these measurements. In both (**A**,**B**), the x-axis indicates particle diameter in nanometers and y-axis indicates particle concentration/ml/nm. Concentrations of particles are indicated above the horizontal line with ± counting error.

To establish if *A. phagocytophilum*-DC (*Ap*-DC) can be used to generate infected ticks, HL-60 cells infected with *A. phagocytophilum* were processed for DC isolation and used for further analysis (Fig. 1). We first measured the concentration and size of host cell-free *A. phagocytophilum*–DC isolated from infected HL-60 cells. We utilized MRPS Spectradyne instrument with TS-2000 cartridge that has a measurement range of ~ 250–2000 nm particle size. Measurements of undiluted *Ap*-DC and PBS control solutions were performed as described in the methods. MRPS spectradyne measurement showed heterogeneously sized population of DC with various sizes ranging from 250 to 1000 nm. Figure 2A shows the MRPS data for *Ap*-DC concentration and size in our preparations. During the measurement, a total of 20,435 particles were analyzed. The total concentration of *Ap*-DC was noted to be 4.73 × 10^8^ ± (3.34 × 10^6^, 3.32 × 10^6^) bacteria/ml. *Ap*-DC that were around size 300–400 nm were more in numbers in comparison to the other sized forms of DC (Fig. 2A). The data also indicated that as the size of *Ap*-DC increased the numbers for those forms also reduced (Fig. 2A). MRPS spectradyne measurements of PBS control solution that was generated from uninfected cells (processed similar way during the preparation of DC from infected cells) showed some background peaks in a size range from 250 to 550 nm (Fig. 2B). A total of 7 different particle sizes were detected in PBS solution with concentration noted to be 7.08 × 10^4^ ± (7.78 × 10^4^, − 2.68 × 10^4^) particles/ml (Fig. 2B). Comparison of particle numbers in *Ap*-DC preparation with PBS control solution indicated that a large number of particles were present in *Ap*-DC preparation (N = 20,435) compared to the PBS (N = 7) control (Fig. 2A,B). The observation of four orders of magnitude of more particles in Ap-DC (N = 20,435) compared to PBS control (N = 7) clearly indicates successful isolation and detection of DC in the preparations. Due to the detection of increased particle numbers in Ap-DC (N = 20,435), the counting error in this sample is close to symmetrical. However, on PBS control, the counting error is much larger in proportion to the measured concentration and very asymmetrical due to less particle numbers (N = 7). The detection of only 7 particles in PBS control over a long period of time could be due to the noise floor for the cartridge used in this measurement. The distribution of excluded and included particles/events during *Ap*-Dc measurement is shown in Supplementary Fig. S1. These data indicated that possibly *Ap*-DC exists more in 300–400 nm size form compared to other sized forms.

### PCR amplification of *A. phagocytophilum* transcripts from *Ap*-DC isolated from HL-60 cells

**Figure 3:**
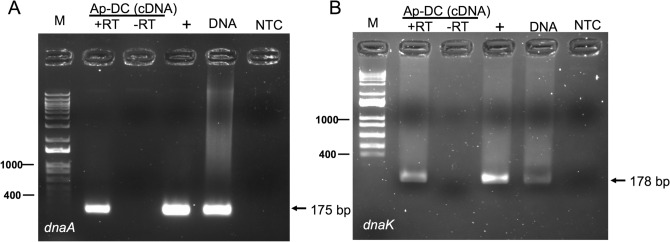
*Ap*-DC forms isolated from HL-60 cultures are viable. Agarose gel electrophoresis image showing PCR amplification of *A. phagocytophilum dnaA* (**A**) or *dnaK* (**B**) products from RNA or DNA samples generated from *Ap*-DC preparations. In both panels, PCR products from RT-PCR reactions performed with cDNA prepared from *Ap*-DC RNA and reverse transcriptase enzyme are denoted as +RT and reactions performed without reverse transcriptase enzyme are denoted as −RT. M indicates DNA ladder marker (1kp plus ladder, Invitrogen). Previously sequenced PCR product was used as a positive control and indicated as +. NTC indicates no template control. DNA generated from the same *Ap*-DC preparation used for RNA isolation was also used in these analyses and is indicated as DNA on the agarose gels in both A and B images.

To test if *Ap*-DC isolated using sonication and differential centrifugation method contain detectable bacterial transcripts, we processed the sample for RNA extraction followed by cDNA synthesis. In addition, DNA was extracted from the same sample. The generated cDNA was used in a RT-PCR to detect *A. phagocytophilum dnaA* and *dnaK* transcripts using the oligonucleotides mentioned in Table S1. Reactions performed in the absence of reverse transcriptase enzyme (− RT) reaction were used as negative controls. The results revealed presence of both *dnaA* and *dnaK* transcripts or gene fragments in RNA or DNA samples generated from same *Ap*-DC preparation, respectively. In the presence of reverse transcriptase enzyme (+RT) expected band at 175 bp for *dnaA* (Fig. 3A) and 178 bp for *dnaK* (Fig. 3B) was noted in RNA samples. Similar sized bands were noted in DNA samples extracted from *Ap*-DC preparation (Fig. 3A,B). No bands were evident in the absence of reverse transcriptase (−RT) enzyme and in no template controls (Fig. 3A,B). Previously sequenced fragments generated from DNA samples of *Ap*-DC were used as a positive control in these PCR reactions (Fig. 3A,B). The detection of *dnaA* and *dnaK* transcripts indicates that *Ap*-DC isolated from *A. phagocytophilum*-infected HL-60 contains amplifiable bacterial mRNA.

### Microinjected unfed nymphal ticks with *Ap*-DC could transmit *A. phagocytophilum* to naïve mammalian host

**Figure 4:**
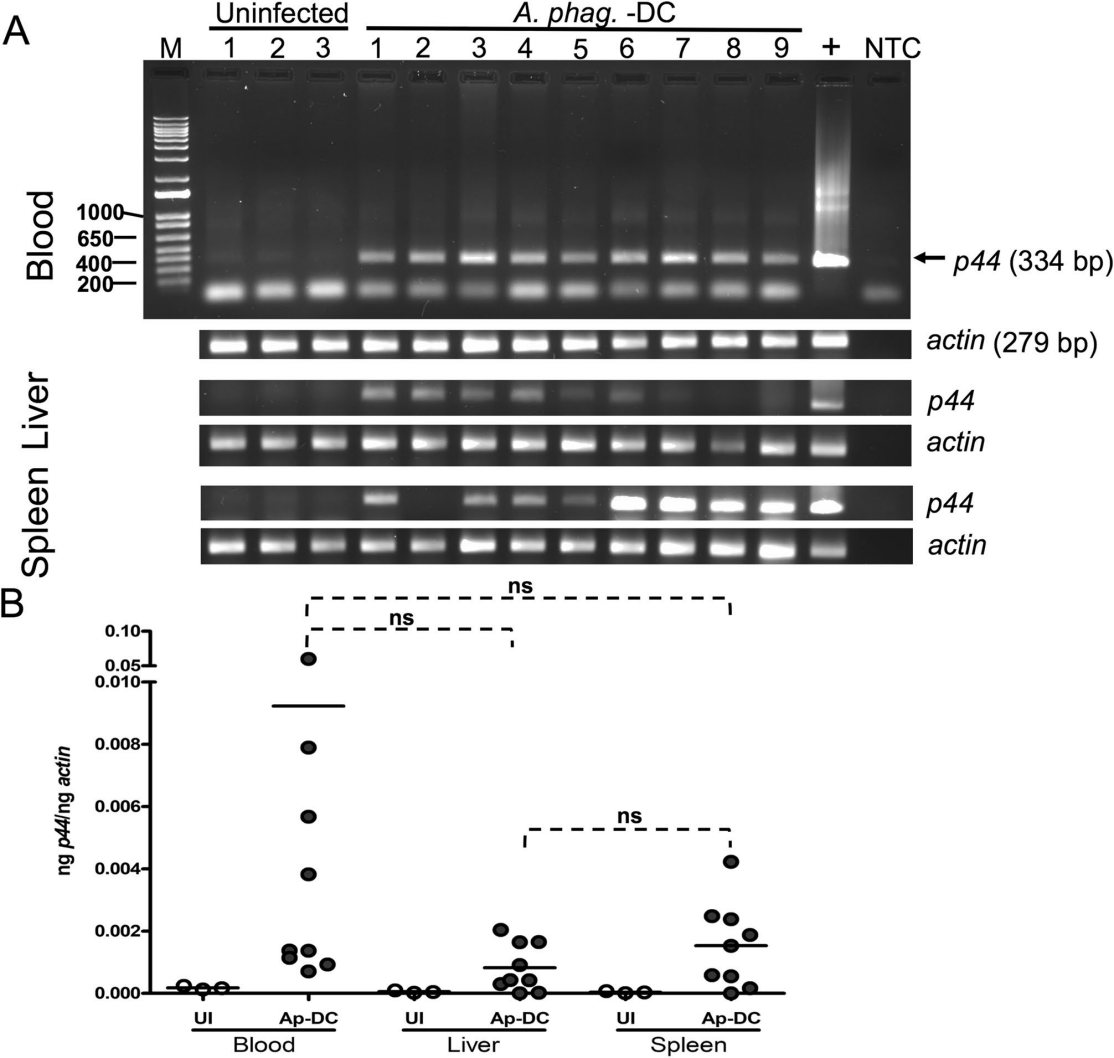
*Ap*-DC microinjected ticks successfully transmit *A. phagocytophilum* to mice. (**A**) Agarose gel electrophoresis image showing PCR amplification of *A. phagocytophilum p44* (size 334 bp) in DNA samples generated from blood, liver and spleen tissues of mice that were fed with *Ap*-DC microinjected nymphal ticks. PCR amplification of mice beta-actin (size 279 bp) was used as a sample control. (**B**) Quantitative RT-PCR (QRT-PCR) analysis showing *A. phagocytophilum p44* loads in DNA samples of mice blood, liver and spleen tissues of mice that were infected via feeding with *Ap*-DC microinjected nymphal ticks. Levels of *A. phagocytophilum p44* were normalized to tick beta-actin levels. Open circles represent data from DNA samples of murine blood, liver and spleen tissues of naïve mice and closed circles represent data from DNA samples generated from mice that were fed with *Ap*-DC-injected nymphs. Statistical significance was calculated using Student’s *t* test and ns indicates not significant. Full-length gel images are shown in Supplementary Figs. S2–S4.

Next, we then analyzed whether the microinjected ticks (generated by microinjection of *Ap*-DC into anal pore of nymphal ticks) could transmit *A. phagocytophilum* to uninfected murine host. First, initial *Ap*-DC preparation (~ 4.7 × 10^8^ bacteria/ml) was 1:100 diluted generating ~ 4.7 × 10^6^ bacteria/ml suspension. This suspension was used for microinjection into unfed nymphal ticks. Our initial attempts with injection suspension taken from ~ 4.7 × 10^8^ bacteria/ml solution to inject *Ap*-DC into tick body were unsuccessful due to higher death observed in ticks. Therefore, we microinjected ~ 4.2 nl of this solution per nymphal tick at its anal pore. Microinjected ticks were allowed to recover for 7 days to facilitate *A. phagocytophilum* colonization and establishment in these ticks. Approximately 90% of ticks survived microinjection and were viable after seven days of incubation. Approximately, 20 microinjected ticks were allowed to feed on each naïve mice. However, we noted that 8–10 microinjected ticks successfully fed on each mouse. Repleted ticks were collected and stored at − 20 °C and processed for DNA extractions as described in methods. After 12 days post tick placement, mice were euthanized, and blood and other tissues such as spleen and liver were harvested. DNA was extracted from mice blood, and tissue samples. PCR analysis revealed detection of *A. phagocytophilum p44* gene fragment (band size = 334 bp) in all nine mice blood samples (Fig. 4A). Nucleotide sequencing of purified PCR products confirmed that the amplified band corresponds to *A. phagocytophilum* DNA. As expected, no bands in three uninfected mice blood samples were noted (Fig. 4A). PCR reactions performed with mice-beta actin oligonucleotides showed the presence of mice DNA (band size = 279 bp) fragment in all tested samples (Fig. 4A, Supplementary Fig. S2). We also tested the presence of *A. phagocytophilum* in liver tissues (Fig. 4A, Supplementary Fig. S3). In DNA samples extracted from liver tissues, *A. phagocytophilum p44* gene fragment was amplified in six out of nine mice liver samples (samples 1–6). Two samples showed a faint amplification (samples 7 and 9), and one sample showed no amplification (sample 8) for *A. phagocytophilum* p44 gene. Similarly, DNA samples from spleen tissues were analyzed (Fig. 4A, Supplementary Fig. S4). We noted that in eight out of nine mice spleen DNA samples (Fig. 4A, Supplementary Fig. S4, samples 1 and 9) clear amplification of *A. phagocytophilum* p44 gene fragment was evident. One sample showed no amplification (Fig. 4A, Supplementary Fig. S4). QRT-PCR further confirmed the presence of *A. phagocytophilum* in blood, liver, and spleen tissues (Fig. 4B). However, no statistically significant difference in the bacterial load was noted between blood and other tested tissues (Fig. 4B). These results show that *Ap*-DC-microinjected unfed nymphal ticks successfully feed and transmit *A. phagocytophilum* to murine host.

### *Ap*-DC-microinjected nymphal ticks retain *A. phagocytophilum* after feeding on murine host

**Figure 5:**
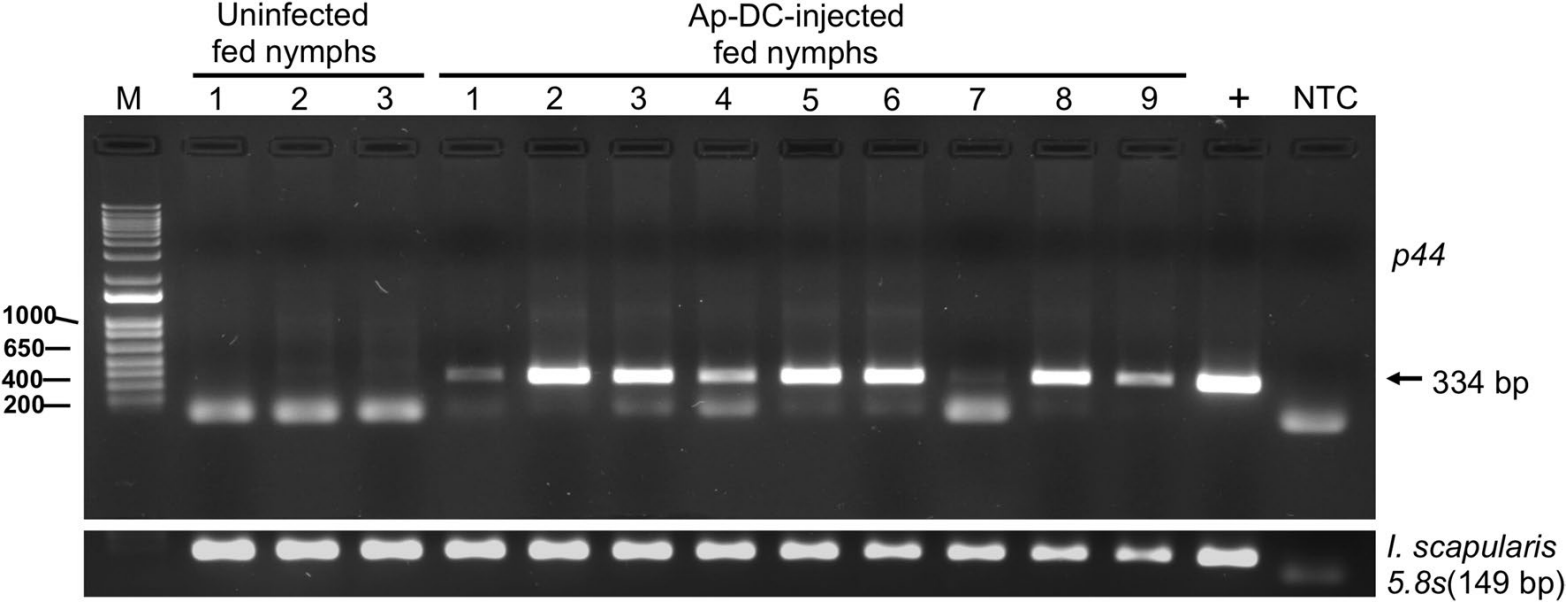
*Ap*-DC-injected nymphal ticks retain bacteria after transmission to murine host. Agarose gel electrophoresis image showing PCR amplification of *A. phagocytophilum p44* gene product from DNA samples generated from *Ap*-DC-injected fed nymphs. Amplification of tick 5.8s rRNA in the same samples was used as sample control. Full length image for tick 5.8s rRNA is shown in Supplementary Fig. S6. M indicates DNA ladder marker (1 kp plus ladder, Invitrogen). PCR amplification of *p44* or 5.8s rRNA in three DNA samples generated from uninfected nymphs fed on naive mice (uninfected fed nymphs) and nine DNA samples from *Ap*-DC-injected nymphs fed on naive mice (*Ap*-DC-injected fed nymphs) is shown. Previously sequenced PCR product (denoted as +) was used as a positive control. NTC indicates no DNA template control.

We then tested if *Ap*-DC microinjected ticks transmit all bacteria after feeding on mice and/or retain some in its body. PCR analysis on individual ticks revealed that out of 20 fed nymphs analyzed, 14 of them showed amplification of *A. phagocytophilum p44* gene fragment (Fig. 5, Supplementary Fig. S5). Nucleotide sequencing of the PCR product confirmed the presence of *A. phagocytophilum* DNA in these ticks. The amplification of tick 5.8s ribosomal RNA served as a sample control in these analyses (Fig. 5, Supplementary Figs. S5, S6). These results show that even though *Ap*-DC-microinjected ticks successfully transmitted *A. phagocytophilum* to the murine host, a majority number of ticks still retained these bacteria in its body.

### Larvae fed on mice that were previously infected with *Ap*-DC-microinjected nymphal ticks successfully acquire *A. phagocytophilum*

**Figure 6:**
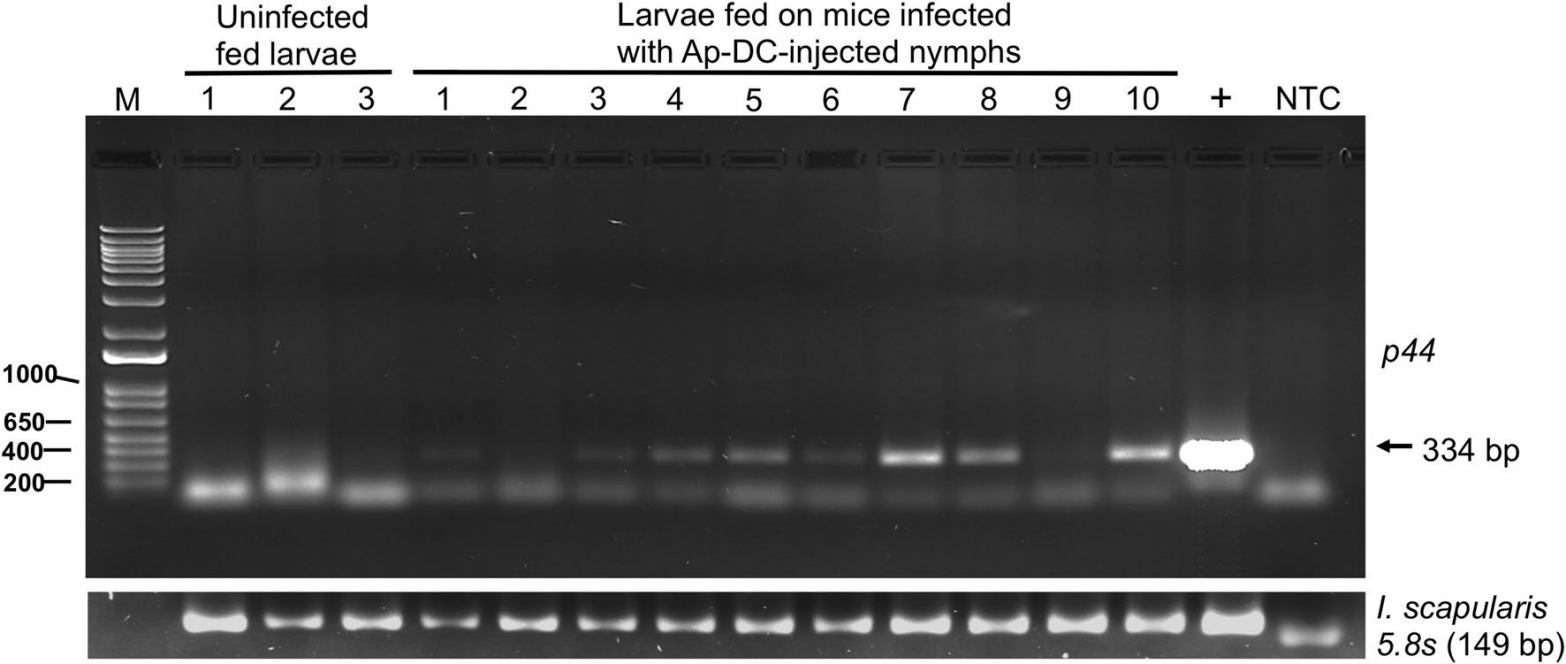
Larvae successfully acquire *A. phagocytophilum* upon feeding on mice previously infected with *Ap*-DC-injected nymphs. Agarose gel electrophoresis image showing PCR amplification of *A. phagocytophilum p44* gene product from DNA samples generated from larvae fed on mice previously infected with *Ap*-DC-injected nymphs. Amplification of tick 5.8s rRNA in the same samples was used as sample control. Full length image for 5.8S rRNA is shown in Supplementary Fig. S8. M indicates DNA ladder (1kp plus ladder, Invitrogen). PCR amplification of *p44* or 5.8s rRNA in DNA samples generated from uninfected larvae fed on naive mice (three samples) and DNA samples from larvae fed on mice previously infected with *Ap*-DC-injected nymphs (nine samples) is shown. Previously sequenced PCR product (denoted as +) was used as a positive control. NTC indicates no template control.

We then determined, if larvae can successfully acquire *A. phagocytophilum* from the murine host that was previously infected with *Ap*-DC-microinjected nymphal ticks. Larvae were fed on C3H/HeN mice that were previously infected with *Ap*-DC-microinjected nymphal ticks. Repleted fed larval ticks were collected and individually processed for DNA extractions as described in methods. PCR analysis revealed amplification of *A. phagocytophilum p44* gene fragment in 19 out of 21 larvae analyzed (Fig. 6, Supplementary Fig. S7). Nucleotide sequencing of the purified PCR products confirmed the presence of *A. phagocytophilum* DNA in these fed larvae. However, two of the samples did not show clear amplification of *A. phagocytophilum p44* gene fragment (Fig. 6). PCR amplification of *I. scapularis* 5.8s ribosomal RNA (Fig. 6, Supplementary Figs. S7, S8) was used as a sample control for this experiment.

### Transstadial transmission of *A. phagocytophilum* from larval to nymphal stage in ticks fed on mice infected with *Ap*-DC-microinjected ticks

**Figure 7:**
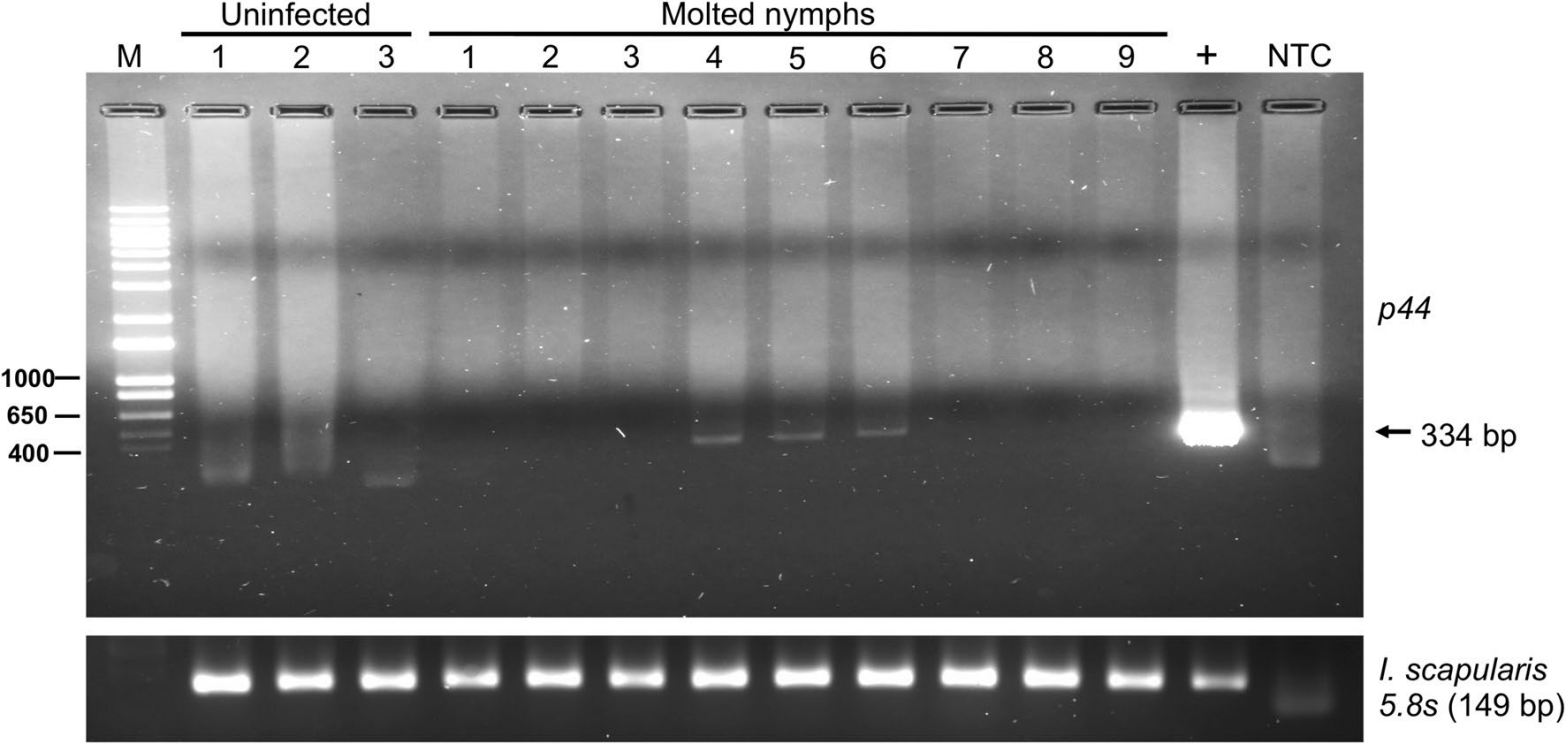
Transstadial transmission of *A. phagocytophilum* from larvae (that were fed on mice infected with *Ap*-DC-injected nymphs) to the next developmental stage. Agarose gel electrophoresis image showing PCR amplification of *A. phagocytophilum p44* gene product in DNA samples of nymphs that were molted from larvae fed on mice infected with *Ap*-DC-injected nymphs. Amplification of tick 5.8s rRNA in the same samples was used as sample control. M indicates DNA ladder marker (1kp plus ladder, Invitrogen). PCR amplification of *p44* or 5.8s rRNA in DNA samples generated from uninfected nymphs molted from larvae fed on naive mice (three samples) and DNA samples from nymphs molted from larvae fed on mice previously infected with *Ap*-DC-injected nymphs (nine samples) is shown. Full length image for 5.8S rRNA is shown in Supplementary Fig. S9. Previously sequenced PCR product (denoted as +) was used as a positive control. NTC indicates no template control.

We then analyzed if larvae fed on mice infected with *Ap*-DC-microinjected ticks could retain *A. phagocytophilum* after molting into next development stage. A group of larvae were therefore allowed to molt to nymphal stage. Freshly molted nymphs were individually processed for DNA extractions and analyzed by PCR for the presence of *A. phagocytophilum.* PCR analysis revealed the presence of *A. phagocytophilum* in three out nine freshly molted nymphs (Fig. 7) indicating successful transstadial transmission of this bacterium from larval to nymphal stage. In addition, nucleotide sequencing of the purified PCR products confirmed the presence of *A. phagocytophilum* DNA in molted nymphs. Amplification of tick 5.8s rRNA fragment was evident in all samples (Fig. 7, Supplementary Fig. S9). DNA from uninfected nymphs that were molted from larvae fed on uninfected mice was used as a control in this analysis. As expected, no *A. phagocytophilum* specific PCR amplified product was observed in larvae fed on uninfected mice (Fig. 7). These results elucidate that larvae fed on mice infected with *Ap*-DC-microinjected ticks could transstadially transmit *A. phagocytophilum* into the next developmental stage.

## Discussion

Hard ticks such as *I. scapularis* requires several days to feed on an animal to acquire or transmit pathogens^7^. Therefore, development of an alternative way of infection in this species would rapidly advance research in studying tick-pathogen interactions. Several artificial membrane-feeding methods have been developed for both hard and soft ticks for the generation of infected ticks^39,40^. Even though these methods are simpler and cost effective, generation of tick cohorts with equal number of pathogens in each tick is limited. The success of anal pore microinjection system to generate Lyme disease pathogen *B. burgdorferi*-infected ticks^41^ or tick-borne encephalitis virus family Langat virus-infected ticks^42^ suggests that this technique can be easily adopted for generating both extracellular or intracellular tick-borne pathogen-infected ticks, respectively. In this study, we report the use of microinjection system to generate obligate intracellular bacterium *A. phagocytophilum*-infected unfed nymphs in the laboratory condition and showed that these ticks can efficiently transmit this bacterium to the murine host.

Quantification of *A. phagocytophilum* by competitive PCR is reported^20^. In addition, electron microscopy methods have been used to count DC isolated from mammalian cells^31^. The competitive PCR method is based upon amount of DNA and electron microscopy method requires multiple microscopic sections to count several and hundreds of DC forms. This study reports the use of Spectradyne system as a novel method to evaluate *Ap-*DC numbers from HL-60 cells. Even though competitive PCR and electron microscopy methods can be used to evaluate *A. phagocytophilum* concentration in different cells, our study shows efficiency on the use of Spectradyne system to count and also analyze sizes of DC forms in a rapid way. A previous study has reported findings from in vitro cell culture experiments and has provided microscopic evidence that within one to four hour post *Anaplasma* infection of tick cells, bacteria attaches to tick plasma membrane and encloses inside an endosome^30^. By eight hours post infection, evidence of possible first bacterial division was observed^30^. At 12 h p.i., appearance of morulae-like shaped inclusions were observed and by 48 h p.i. endosomes harboring large numbers of *Anaplasma* were noted^30^. At 72 h p.i. highly pleomorphic sizes of *Anaplasma* was observed^30^. The observation of heterogeneous population of DC in the range from 300 to 1200 nm noted from Spectradyne measurements in this study corroborates with the reported microscopic observations from the previous study^30^. This method not only provides quantitative information on Ap-DC numbers but also provides quantitative information on the size of Ap-DC forms. Compared to the other existing methods, we believe that by using Spectradyne measurement one could rapidly quantitate both numbers and size of Ap-DC in samples.

Our study indicates that using this method, we can rapidly and efficiently generate *A. phagocytophilum*-infected unfed nymphs in the laboratory conditions. In nature, both *I. scapularis* nymphs and adults could transmit tick-borne pathogens to humans^7^. Several studies, including our own, have characterized several tick molecules in *A. phagocytophilum*-nymphal *I. scapularis* interactions^22–26^. However, studies in understanding *A. phagocytophilum*-adult tick interactions are limiting. We believe that the method reported in this study could be easily adapted to directly inject DC into adult *I. scapularis* to generate *A. phagocytophilum*-infected unfed adult ticks. Characterization of bacterial and/or tick molecules in adult ticks would provide new information in understanding *A. phagocytophilum*-adult tick interactions. The observation of bacterial loads in only 3 out of 9 freshly molted nymphs suggests that bacterial loads could be very low and are at undetectable levels in some of the nymphs. In addition, the observation of increased death in nymphs upon initial injection with undiluted stock (injection suspension taken from ~ 4.7 × 10^8^ bacteria/ml solution) suggests that ticks cannot tolerate high dose of bacteria. Future experiments can be performed with different doses of DC to determine lethal dose of 50% (LD_50_) for ticks. Tick molecules have been considered as candidates for the development of transmission-blocking vaccines^43^. Therefore, availability of methods, such as this one, to generate unfed *A. phagocytophilum*-infected unfed nymphal or adult ticks would facilitate rapid testing on the effect of active or passive immunization strategies (developed against tick molecules) to block transmission of *A. phagocytophilum* from vector to the vertebrate host.

The success in the transmission of *A. phagocytophilum* from *I. scapularis* that were microinjected with DC into anal pore of unfed ticks is not surprising. In the natural acquisition route, *A. phagocytophilum* migrates from an infected animal to the gut of an engorging tick through a blood meal^2,20^. The bacterium then enters tick gut cells and undergoes first round of replication^2,20^. Later, *A. phagocytophilum* migrate and colonize tick salivary gland cells^2,20^. In the natural transmission route, *A. phagocytophilum* that are colonized in tick salivary glands cells, are transmitted to the vertebrate host along with saliva during blood feeding^2,20^. As anal pore connects to the gut, we believe that injection of *Ap*-DC into this provides a partial/artificial acquisition route for this bacterium similar to its natural route to enter tick gut first, undergo initial replication and then colonize in tick salivary glands. Incubation of *Ap*-DC-microinjected ticks for seven days may additionally facilitate replication and establishment of *A. phagocytophilum* in ticks. Even though we observed increased trend in bacterial loads in samples generated from murine blood compared to levels noted in samples generated from other tissues, we did not noted statistical difference between them. This could be due to high variability in bacterial numbers between murine tissue samples. The successful transmission of *A. phagocytophilum* from *Ap*-DC-injected ticks to murine host suggests that this method could also serve as an alternative method to generate a natural transmission route environment for this bacterium.

Collectively, we describe a timely, rapid and an efficient microinjection method to generate *A. phagocytophilum*-infected ticks in the laboratory. Studies like this would substantially enhance research not only in understanding this and perhaps other rickettsial pathogen-tick interactions but could also accelerate anti-vector/transmission-blocking vaccine research in the field of tick-borne diseases.

## Supplementary information

Supplementary Information.

## Abbreviations

RC: Reticulate cells
DC: Dense-core form
*Ap*-DC: *A. phagocytophilum* Dense-core form
p.i.: Post infection
HL-60: Human leukemia cell line
PBS: Phosphate-buffered saline
