# Supplementary material for: An efficient microinjection method to generate human anaplasmosis agent Anaplasma phagocytophilum-infected ticks
Source: Sci Rep. 2020 Sep 29;10:15994. doi: 10.1038/s41598-020-73061-9 (PMC7524789; doi:10.1038/s41598-020-73061-9)
Supplement: Supplementary file 1 — Supplementary Information. [file 41598_2020_73061_MOESM1_ESM.pdf]

An efficient microinjection method to generate human anaplasmosis agent *Anaplasma phagocytophilum*-infected ticks

Vikas Taank [1], Ellango Ramasamy [1], Hameeda Sultana [1, 2], and Girish Neelakanta [1, 2, $]

Supplementary information

Supplementary Figure legends

Supplementary Figure 1. MRSP spectradyne measurement of *Ap*-DC. Microfluidics resistive pulse sensing (MRPS) using Spectradyne instrument (nCS1, Spectradyne LLC, USA) was used to determine the size distribution and concentration of isolated *A. phagocytophilum* -DC. Diameter versus transit time is shown. TS-2000 cartridge was used to perform the measurements. The graph shows the excluded (blue dots) and included (red dots) events during the MRPS measurements for *Ap*-DC. The X-axis shows the transit time (μs), and Y-axis shows the particle diameter (nm).

Supplementary Figure 2. Full images of PCR analysis for actin in mice blood samples. Full image of PCR amplification of mouse beta-actin fragment in blood samples is shown. This data is shown as a cropped image in main Figure 4A.

Supplementary Figure 3. Full images of PCR analysis for *A. phagocytophilum* p44 and mouse beta-actin in murine liver samples. Full image of PCR amplification of *A. phagocytophilum* p44 and mouse beta-actin fragments in liver samples is shown. This data is shown as a cropped image in main Figure 4A.

Supplementary Figure 4. Full images of PCR analysis for *A. phagocytophilum* p44 and mouse beta-actin in murine spleen samples. Full image of PCR amplification of *A. phagocytophilum* p44 and mouse beta-actin fragments in spleen samples is shown. This data is shown as a cropped image in main Figure 4A.

Supplementary Figure 5. Analysis of additional *Ap*-DC-injected nymphal ticks that retain bacteria in its body after feeding on a vertebrate host. Agarose gel electrophoresis image showing PCR amplification of *A. phagocytophilum p44* gene product from DNA samples generated from *Ap*-DC-injected fed nymphs. Amplification of tick 5.8s rRNA in the same samples was used as sample control. M indicates DNA ladder marker (1kp plus ladder, Invitrogen). PCR amplification of *p44* or 5.8s rRNA in three DNA samples generated from uninfected nymphs fed on naive mice (uninfected fed nymphs) and eleven DNA samples from *Ap*-DC-injected nymphs fed on naive mice (*Ap*-DC-injected fed nymphs) is shown. Previously sequenced PCR product (denoted as +) was used as a positive control. NTC indicates no DNA template control.

Supplementary Figure 6. Full images of PCR analysis for *I. scapularis 5.8S rRNA* in DC-injected fed nymphs. Full image of PCR amplification of *I. scapularis 5.8S rRNA* fragment in DC-injected fed tick samples is shown. This data is shown as a cropped image in main Figure 5.

Supplementary Figure 7. Analysis of additional larvae that shows successful acquisition of *A. phagocytophilum* from mice previously infected with *Ap*-DC-injected nymphs. Agarose gel electrophoresis image showing PCR amplification of *A. phagocytophilum p44* gene product from DNA samples generated from larvae fed on infected mice (generated by feeding *Ap*-DC-injected nymphs). Amplification of tick 5.8s rRNA in same samples was used as sample control. M indicates DNA ladder marker (1kp plus ladder, Invitrogen). PCR amplification of *p44* or 5.8s rRNA in DNA samples generated from uninfected larvae fed on naive mice (three samples) and DNA samples from larvae fed on mice previously infected with *Ap*-DC-injected nymphs (eleven samples) is shown. Previously sequenced PCR product (denoted as +) was used as a positive control. NTC indicates no DNA template control.

Supplementary Figure 8. Full images of PCR analysis for *I. scapularis 5.8S rRNA* in larvae fed on mice infected with DC-injected nymphs. Full image of PCR amplification of *I. scapularis 5.8S rRNA* fragment in larvae fed on mice infected with DC-injected nymphs is shown. This data is shown as a cropped image in main Figure 6.

Supplementary Figure 9. Full images of PCR analysis for *I. scapularis 5.8S rRNA* in molted nymphs. Full image of PCR amplification of *I. scapularis 5.8S rRNA* fragment in nymphs molted from larvae fed on mice infected with DC-injected nymphs is shown. This data is shown as a cropped image in main Figure 7.

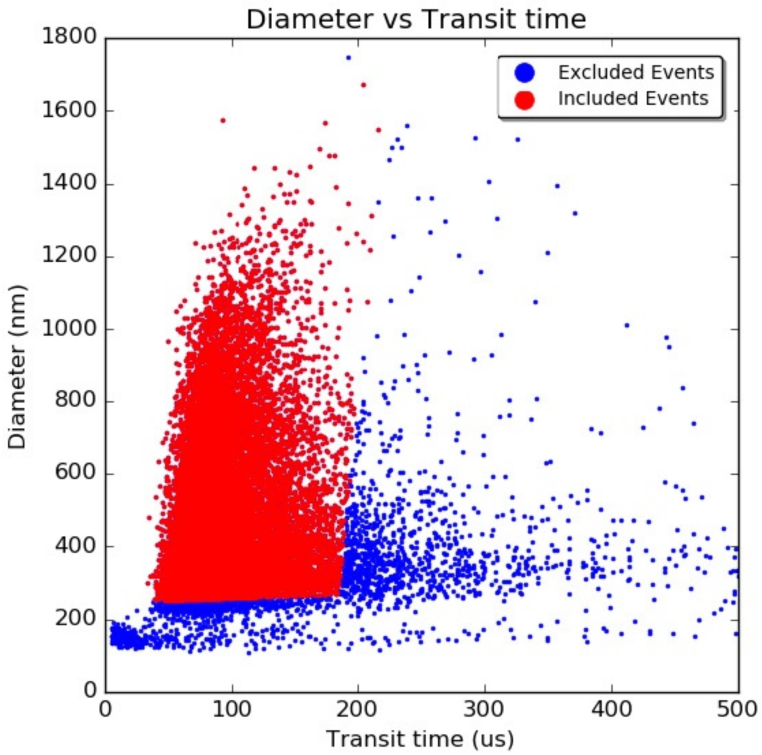

Supplementary Figure 1

Blood Actin

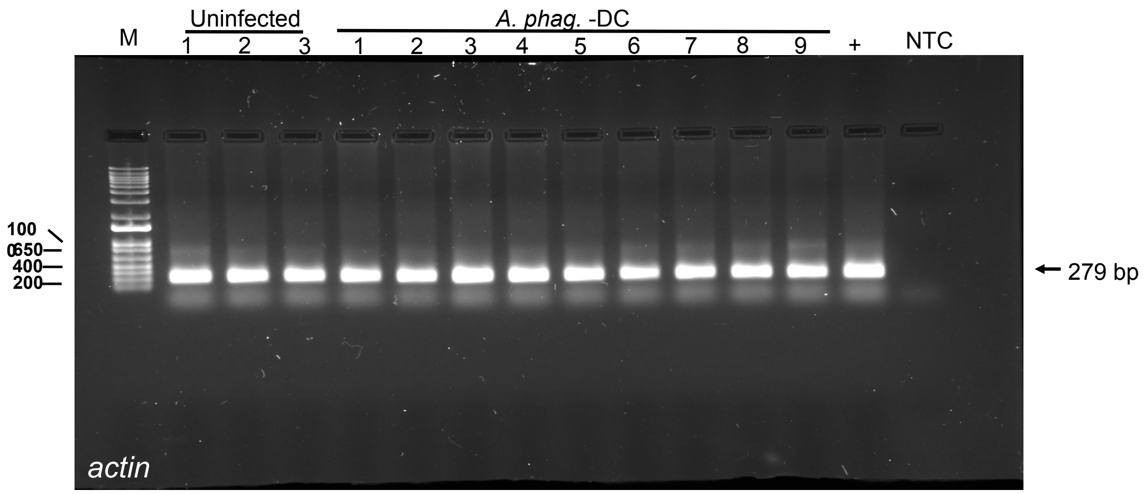

Supplementary Figure 2

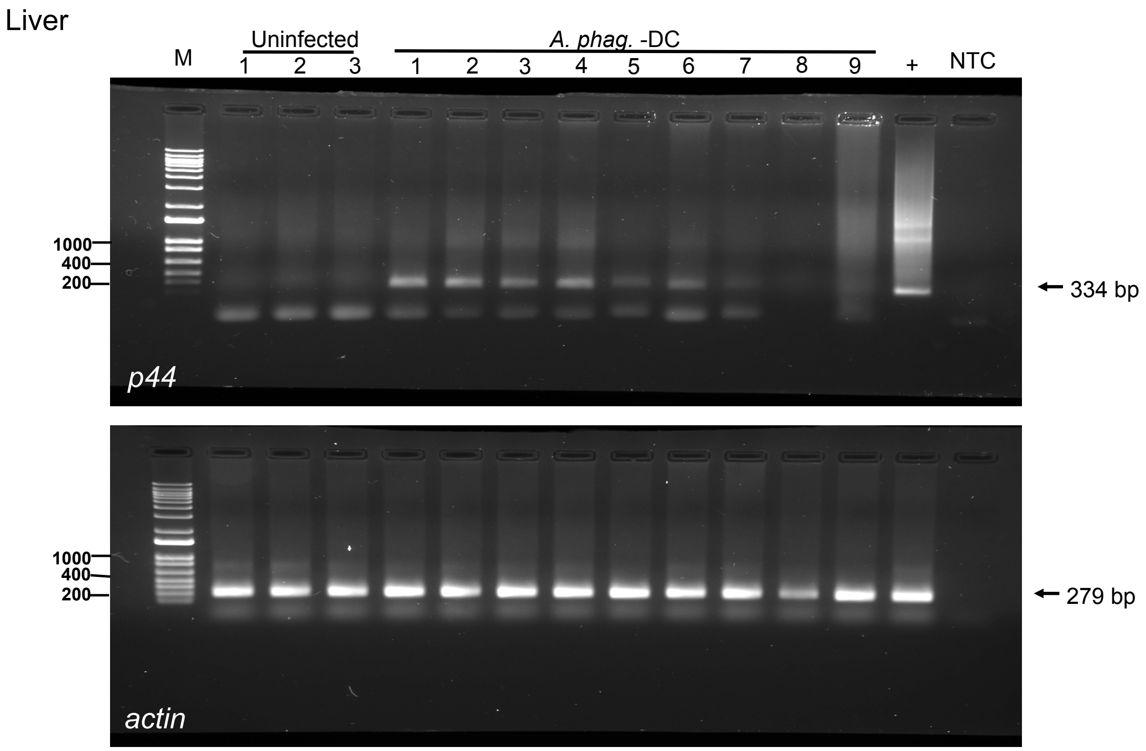

Supplementary Figure 3

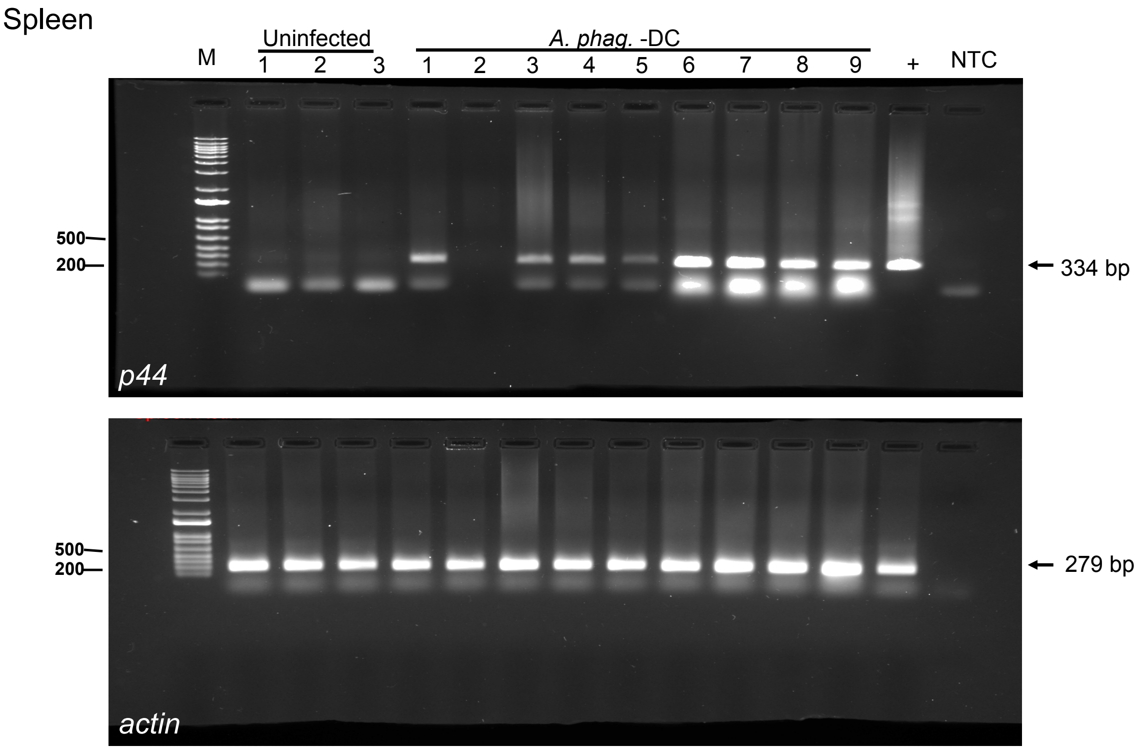

Supplementary Figure 4

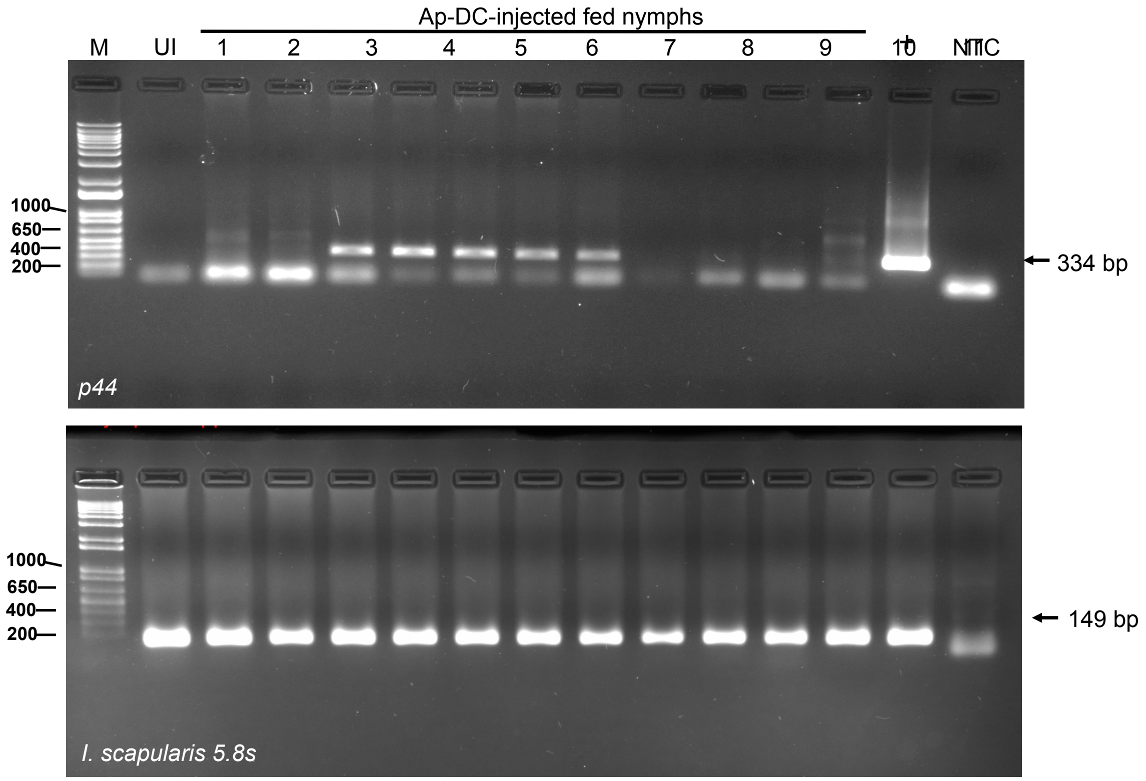

Supplementary Figure 5

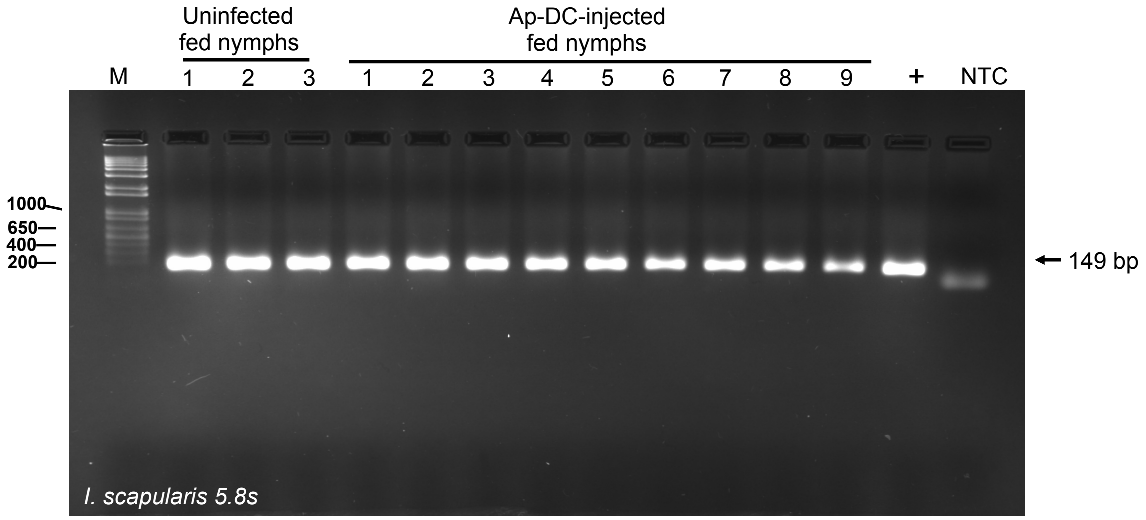

Supplementary Figure 6

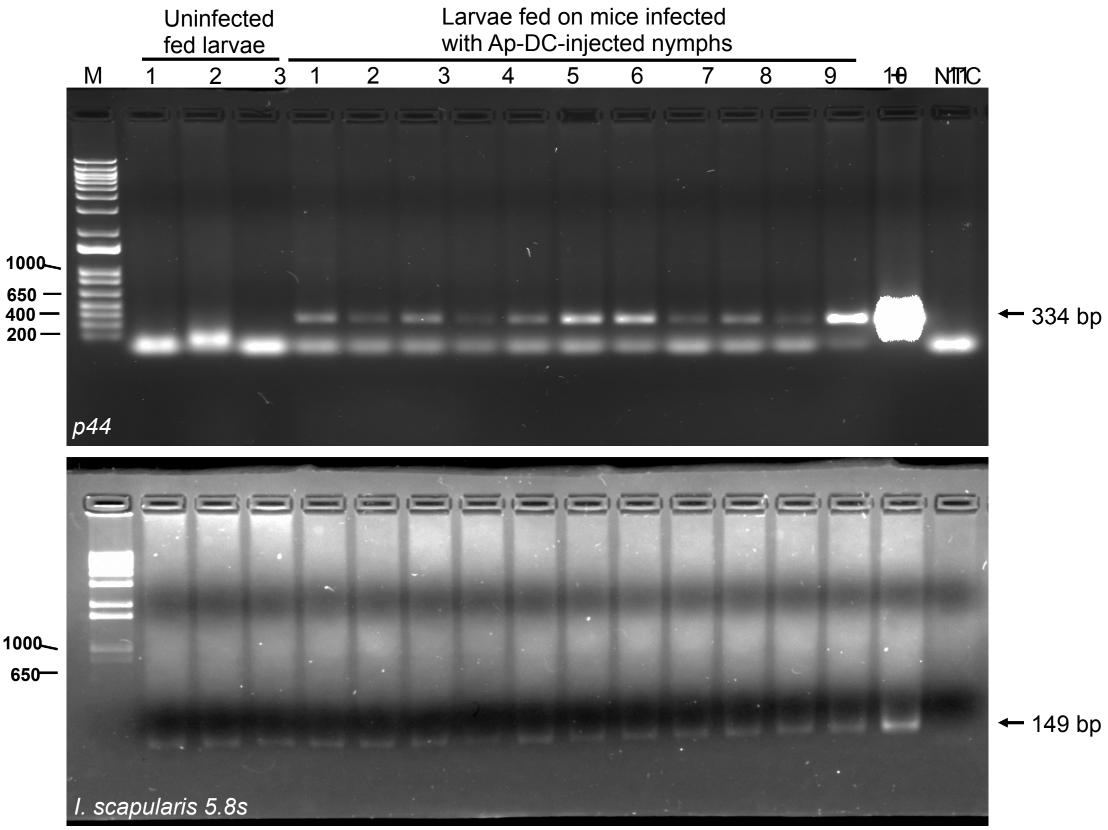

Supplementary Figure 7

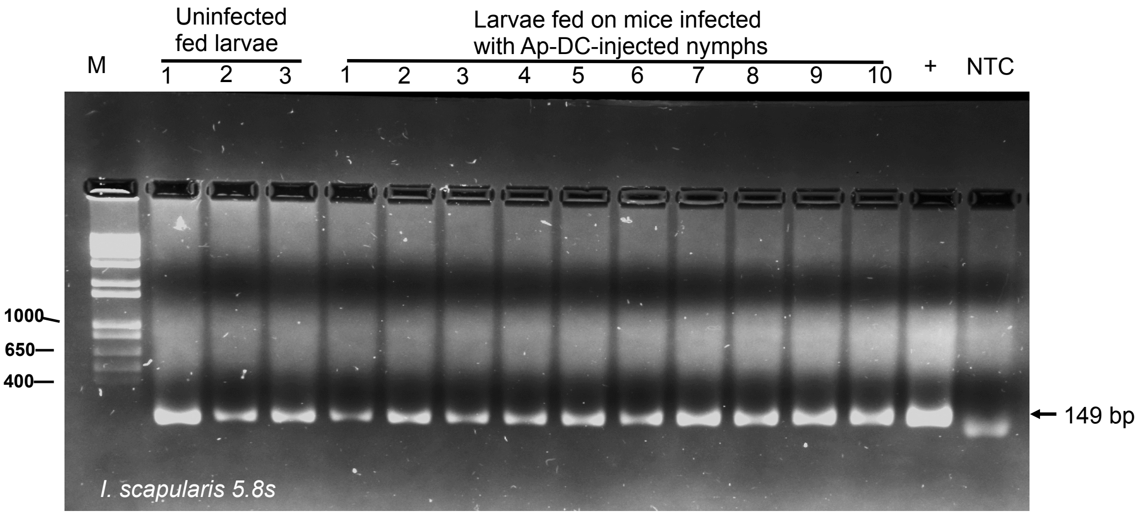

Supplementary Figure 8

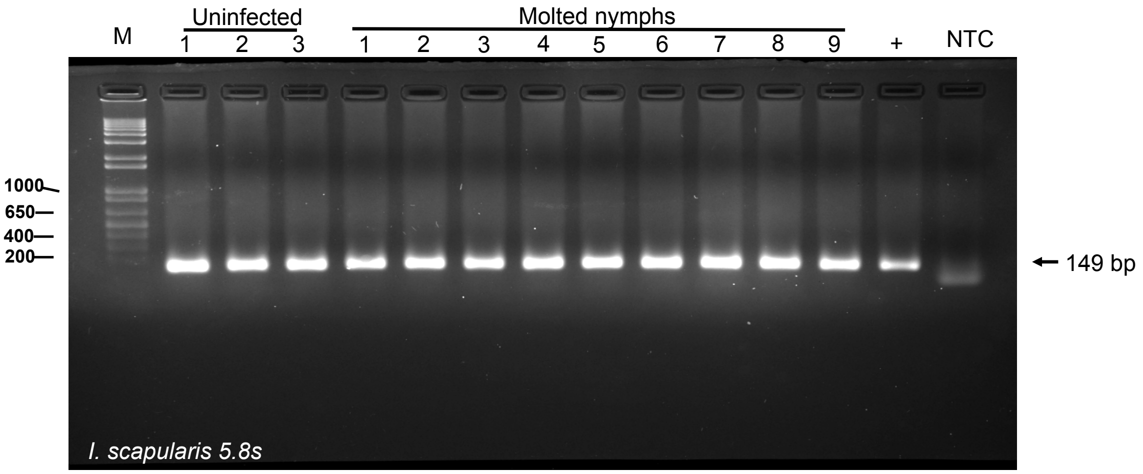

Supplementary Figure 9

Table S1. List of oligonucleotides used in this study.

Primer (5`-3`)	Gene, purpose
CCAGCGTTTAGCAAGATAAGAG	*Anaplasma* p44, qpcr, pcr
GCCCAGTAACAACATCATAAGC	*Anaplasma* p44, qpcr, pcr
CCCTAGGCACCAGGGTGTGA	mice *actin*, qpcr, pcr
GGGGTGTTGAAGGTCTCAAACA	mice *actin*, qpcr, pcr
TCTAAGCGGTGGATCACTCGGT	tick *5.8s*, qpcr, pcr
GACCCTCACACAGACGAAGCCA	tick *5.8s*, qpcr, pcr
GCGGTTCATAAAGGAGTGGA	*Anaplasma dnaA*, qpcr (GenBank acc. no. LANT01000003)
AAACTTCCTGCGCCTCTGTA	*Anaplasma dnaA*, qpcr (GenBank acc. no. LANT01000003)
GATTGGCGCGTTTGTCTTGGA	*Anaplasma dnaK*, qpcr (GenBank acc. no. LANT01000002)
CAAAGCTGCTGCTGTAGGTTCA	*Anaplasma dnaK*, qpcr (GenBank acc. no. LANT01000002)
